# Comparative Genome and Evolution Analyses of an Endangered Stony Coral Species *Dendrophyllia cribrosa* Near Dokdo Islands in the East Sea

**DOI:** 10.1093/gbe/evac132

**Published:** 2022-08-26

**Authors:** Jungeun Kim, Jae Pil Choi, Min Sun Kim, Yejin Jo, Won Gi Min, Seonock Woo, Seungshic Yum, Jong Bhak

**Affiliations:** Personal Genomics Institute (PGI), Genome Research Foundation (GRF), Cheongju, Republic of Korea; Personal Genomics Institute (PGI), Genome Research Foundation (GRF), Cheongju, Republic of Korea; Personal Genomics Institute (PGI), Genome Research Foundation (GRF), Cheongju, Republic of Korea; Ecological Risk Research Division, Korea Institute of Ocean Science and Technology (KIOST), Geoje, Republic of Korea; Ulleungdo-Dockdo Ocean Science Station, KIOST, Ulleung, Gyeongbuk, Republic of Korea; Marine Biotechnology Research Center, KIOST, Busan, Republic of Korea; Ecological Risk Research Division, Korea Institute of Ocean Science and Technology (KIOST), Geoje, Republic of Korea; The KIOST School, University of Science and Technology (UST), Geoje, Republic of Korea; Personal Genomics Institute (PGI), Genome Research Foundation (GRF), Cheongju, Republic of Korea; Korean Genomics Center (KOGIC), Ulsan National Institute of Science and Technology (UNIST), Ulsan, Republic of Korea; Department of Biomedical Engineering, School of Life Sciences, UNIST, Ulsan, Republic of Korea; Clinomics, Inc., Ulsan, Republic of Korea

**Keywords:** *Dendrophyllia cribrosa*, comparative genome, comparative evolution, stony coral, chromosome-level assembly

## Abstract

Stony corals often harbor intracellular photosynthetic dinoflagellate algae that receive dissolved inorganic nutrients. However, *Dendrophyllia cribrosa* is a nonsymbiotic stony coral distributed in the western Pacific. We assembled a chromosome-level *D. cribrosa* genome using PacBio and Hi-C technologies. The final assembly was 625 Mb, distributed on 14 chromosomes, and contained 30,493 protein-coding genes. The Benchmarking Universal Single-Copy Orthologs analysis revealed a percentage of 96.8 of the metazoan genome. A comparative phylogenetic analysis revealed that *D. cribrosa*, which lacks symbionts, evolved to acquire cellular energy by expanding genes related to acyl-CoA metabolism and carbohydrate transporters. This species also has expanded immune-related genes involved in the receptor protein tyrosine kinase signaling pathway. In addition, we observed a specific expansion of calcification genes, such as *coral acid-rich proteins* and *carbonic anhydrase*, in *D. cribrosa*. This high-quality reference genome and comparative analysis provides insights into the ecology and evolution of nonsymbiotic stony corals.

**Significance**

*Dendrophyllia cribrosa* is a nonsymbiotic stony coral. We first provide a chromosome-level genome assembly of *D. cribrosa*, which has a size of 625 Mb forming 14 chromosomes. Our comparative analysis reveals a larger proportion of genes associated with acyl-CoA metabolism and carbohydrate transporters. We also find an expansion of the calcification-related genes. These results provide new insights into the metabolism of these stony corals which lack any symbiont.

## Introduction

**Fig. 1.**
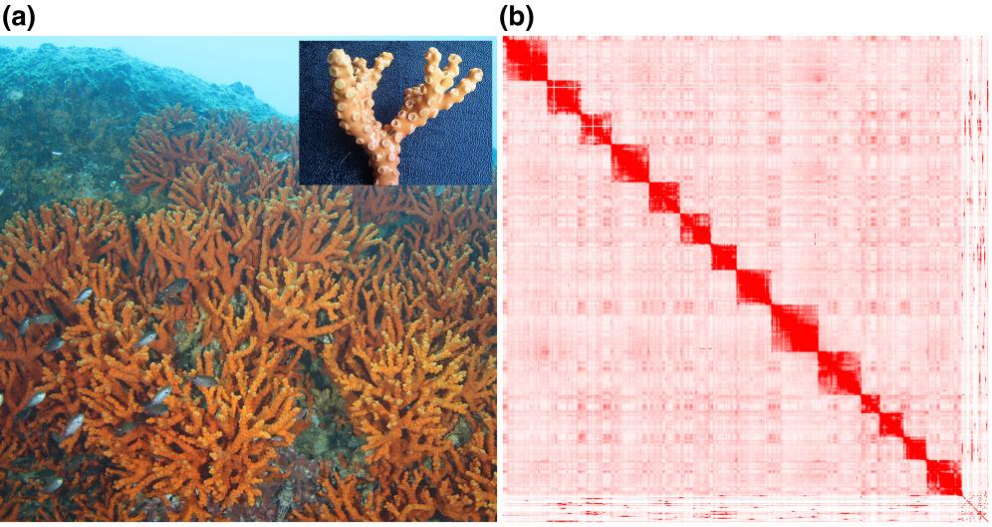
*Dendrophyllia cribrosa* close-up image and its chromosome contact map. (*a*) *Dendrophyllia cribrosa* inhabiting the sea near the Dokdo Islands and its close-up image. (*b*) Chromosome contact map of *D. cribrosa*.

*Dendrophyllia cribrosa*, belonging to the scleractinian coral family, is a rare subtropical–temperate coral species that is distributed in the western Pacific. *Dendrophyllia cribrosa* is a stony coral without symbiotic microalgae. In 2016, the Ministry of Oceans and Fisheries of Korea reported a single habitat of a *D. cribrosa* coral community with a width of 5 m and a height of 3 m, at depths of 18–20  m, near the Dokdo Islands in the East Sea. The morphological features of *D. cribrosa* resemble trees with irregular thick branches (fig. 1*a*), and their coloration ranges from deep yellow to orange. This species was designated as endangered in the “Endangered and Protected Wild Species List in Korea” in 1998 by the Korean Government.

Here, we describe a chromosome-level assembly of *D. cribrosa* from the Dokdo Islands in Korea. The *Dendrophyllia cribrosa* genome provides a comparative study of coral genomes that exhibit evolutionary expansions related to coral calcification, metabolism and immune responses.

## Results and Discussion

### Genome Assembly of *Dendrophyllia cribrosa*

We produced 41 Gb next-generation sequencing (NGS) reads of *D. cribrosa* (supplementary table S1, Supplementary Material online). To estimate the genome size of *D. cribrosa*, we used Jellyfish, programmed with a K-mer range of 17–25. Jellyfish estimated the genome size of *D. cribrosa* to be 610 Mb, at *K* = 25, with the lowest PCR error rate (0.19) and PCR duplicates (0.92), which is similar to the genome size of other closely related complex corals (*Montipora* spp. 615–653). We added the GenomeScope result in supplementary figure S1, Supplementary Material online. At *K* = 25, we estimated the *D. cribrosa* genome size to be 610 Mb with 0.30% heterozygosity in 25 bp of K-mer (supplementary fig. S1, Supplementary Material online). This estimation is similar to the genome size of closely related complex corals (Genus *Montipora*, 615–653 Mb) (Helmkampf et al. 2019). We also produced 120 Gb-long reads (∼246-fold coverage of the genome) using a PacBio Sequel2 platform (DNA Link Inc., Seoul, Republic of Korea) with an N50 of 27 kb (supplementary table S2, Supplementary Material online). The FALCON_unzip assembler constructed 1,174 contigs, with an assembly length of 765 Mb (table 1). After implementing purge haplotigs and error correction, we obtained a 680M assembly from 591 contigs. The Benchmarking Universal Single-Copy Orthologs (BUSCO) assessments showed that the number of “complete and duplicated BUSCO genes” was slightly decreased from 23 (9.0%) to 10 (3.9%) without any change in the total number of complete genes. After polishing the haplotigs, we could not find any changes in BUSCO values (table 1). The N50 of our contigs was 2.1 Mb and L50 was 104 Mb. Using 105 Gb Hi-C reads (∼172-fold coverage), we obtained 22 scaffolds with a 627 Mb *D. cribrosa* genome (fig. 1*b*). Our Hi-C scaffolding resulted in relatively clear chromosomal compartments as shown in supplementary figure S2, Supplementary Material online. However, as denoted in the blue boxes (supplementary fig. S2*a*, Supplementary Material online), the results included two erroneously generated pseudo-scaffolds. The pseudo-scaffolds comprised seven scaffolds grouped into one gigantic chromosome-scale scaffold and two scaffolds grouped into a comparatively small chromosome-scale scaffold (supplementary fig. S2*a*, Supplementary Material online). To resolve this, we manually split these pseudo-scaffolds (supplementary fig. S2*b*, Supplementary Material online) and removed seven contigs to generate the separated scaffolds. Additionally, we did not use any contigs that were <1 kb in the BUSCO assessment, which resulted in a lower score (93.7%). The N50 of the *D. cribrosa* assembly was 19 Mb, and the maximum assembly length was 62 Mb. Based on the BUSCO assessment score, we measured 93.7% completeness of genes, including 92.5% completeness of 236 single-copy genes and 1.2% completeness of three duplicated BUSCO genes. We found 14 (0.8%) missing genes in 14 pseudo-chromosomes. During the scaffolding, several genes were not integrated in the scaffolds.

**Table 1:** Statistics of *Dendrophyllia cribrosa* Assembly

	FALCON	Purge Haplotig	Error Correction (Pilon)	Hi-C Assembly
**No. of contigs**	1,174	591	591	14
**Assembly length**	764,923,991	680,856,317	680,577,677	627,238,274
**Longest contigs**	6,877,747	6,877,747	6,877,747	62,021,193
**N50**	1,750,786	2,113,998	2,112,715	48,602,881
**L50**	126	104	104	6
**BUSCO^a^**	C: 98.8% [S: 89.8%, D: 9.0%], F: 0.0%, M: 1.2%, *n*: 255	C: 98.4% [S: 94.5%, D: 3.9%], F: 0.4%, M: 1.2%, *n*: 255	C: 98.4% [S: 94.5%, D: 3.9%], F: 0.4%, M: 1.2%, *n*: 255	C: 93.7% [S: 92.5%, D: 1.2%], F: 0.8%, M: 5.5%, *n*: 255

a BUSCO version: eukaryota_odb10 (10 September 2020); C, complete BUSCOs; S, complete and single-copy BUSCOs; D, complete and duplicated BUSCOs; F, fragmented BUSCOs; M, missing BUSCOs.

Approximately 364 Mb (58.10%) of repeats were found in the *D. cribrosa* genome (supplementary table S3, Supplementary Material online). This proportion is similar to that of other coral genomes, such as *Trachythela* (57.88%) (Zhou et al. 2021). We predicted 30,493 protein-coding genes in the *D. cribrosa* genome from these data. They showed a slightly higher number of protein-coding genes compared with other coral genes (supplementary table S4, Supplementary Material online). We conducted BUSCO assessment in the protein-coding genes. It resulted in 231 (90.6%) complete eukaryote genes, with 226 (88.6%) single copy and 5 (2.0%) duplicated in the BUSCO gene set. Among them, 8 (3.1%) were fragmented and 16 (6.3%) were missed. The BUSCO assessment showed a higher number of complete genes in the *D. cribrosa* genome (supplementary table S4, Supplementary Material online).

## Materials and Methods

### Sample Collections and Genome Sequencing

*Dendrophyllia cribrosa* colonies were collected at 37°14.6498′ N and 131°51.6516′ E at a depth of 18–20  m using SCUBA diving equipment. The colonies were snap-frozen in liquid nitrogen and stored at −75 °C. Total DNA was extracted from a colony of *D. cribrosa* and processed according to a previously described method optimized for marine invertebrates at the Korea Institute of Ocean Science and Technology (KIOST, Geoje, Republic of Korea) (Kim et al. 2019b).

DNA libraries were constructed using a TruSeq Nano HT Sample Preparation Kit (Illumina, San Diego, CA, USA), and paired-end reads were generated on a NovaSeq 6000 (Illumina) according to the manufacturer’s instructions (supplementary table S2, Supplementary Material online). We then removed adaptors and low-quality reads (*Q* < 20) using Trimmomatic (ver. 0.64; RRID: SCR_011848) (Bolger et al. 2014).

A long-read sequence library was constructed using the SMRTbell Express Template Preparation Kit (101-357-000) and sequenced using the PacBio Sequel2 platform. An Arima-Hi-C kit (Arima Genomics Inc., San Diego, CA, USA) was used according to the manufacturer’s instructions. The Hi-C library was sequenced using the NovaSeq 6000 platform (Novogene Co. Ltd, CA, USA).

An Illumina RNA library from *D. cribrosa* was constructed using the Illumina TruSeq Stranded mRNA LT Sample Prep Kit (Illumina, San Diego, CA, USA) and sequenced using the NovaSeq 6000 platform (DNA Link Inc., Seoul, Republic of Korea). Adaptor and low-quality reads (*Q* < 20) were removed using Trimmomatic (ver. 0.39; RRID: SCR_011848) (Bolger et al. 2014).

### Genome Assembly

Using cleaned Illumina reads, we estimated the genome size of *D. cribrosa* using Jellyfish (ver. 2.2.4; RRID: SCR_005491), a tool for fast, memory-efficient counting of K-mers in DNA (Marcais and Kingsford 2011) and GenomeScope (ver. 2; RRID: SCR_017014) (Ranallo-Benavidez et al. 2020) (supplementary fig. S1, Supplementary Material online). We used the Jellyfish program set with a K-mer range of 17–25 at *K* = 25, with the lowest PCR error rate (0.19) and PCR duplicates (0.92). We have added the GenomeScope result in supplementary figure S1, Supplementary Material online. We assembled the *D. cribrosa* genome using the FALCON_unzip assembler (ver. 1.22; RRID: SCR_016089) with default options and raw reads. We also constructed a deduplicated haploid assembly using the Purge Haplotigs (ver.1.1.2; RRID: SCR_017616) (Roach et al. 2018). To polish our assembly, short reads (61× coverage) were aligned to the *D. cribrosa* haplotigs using the Burrows-Wheeler Alignment tool (BWA) (ver. 0.7.17; RRID: SCR_010910) (Li and Durbin 2009) and possible errors were corrected using Pilon (ver. 1.23; RRID: SCR_014731) (Walker et al. 2014). Using 172x Hi-C reads, scaffolding was conducted with Juicer (ver. 1.6; RRID: SCR_017226) and the 3D-DNA pipeline. A total of 14 pseudo-chromosomes were constructed after a manual curation of the assembly using Juicebox Assembly Tools (ver. 1.13.01; RRID: SCR_021172).

To estimate the number of repetitive sequences in the *D. cribrosa* genome, we built a custom-repeat library using RepeatModeler2 (RRID: SCR_015027) (Flynn et al. 2020) and predicted repeats using RepeatMasker (ver. 4.1.0; RRID: SCR_012954) (supplementary table S3, Supplementary Material online). To estimate the number of protein-coding genes in *D. cribrosa*, we assembled the RNA-seq data using Trinity (ver. 2.10.0; RRID: SCR_013048) (Haas et al. 2013) and aligned the transcript assembly with GMAP (downloaded on February 22, 2021; RRID: SCR_008992) (Wu and Watanabe 2005). We also aligned the RNA-seq data to repeat the masked assembly using HISAT2 (ver. 2.2.1) (Kim et al. 2019a). We applied the Gaius-Augustus/BRAKER pipeline (ver. 2.1.5; RRID: SCR_018964) (Lomsadze et al. 2014; Hoff et al. 2016; Bruna et al. 2021) and assembled the transcripts from RNA-seq data using Trinity (ver. 2.10.0; RRID: SCR_013048) (Haas et al. 2013). We used *Acropora millepora* (Ying et al. 2019) and *Acropora acuminata* (Shinzato et al. 2020) protein sequences for alignment by using exonerate (RRID: SCR_016088) (Slater and Birney 2005). These two genomes showed higher BUSCO values of 96.0% and 93.8%, respectively. For de novo gene prediction, we used AUGUTUS (ver. 3.4.0; RRID: SCR_008417), which was trained with RNA-seq data with default options. Finally, we predicted protein-coding genes by integrating evidence sequences with the EVidenceModeler (ver. 1.1.1; RRID: SCR_014659) (Haas et al. 2008).

### Evolutionary Study of the Coral Genomes

We collected nine coral genomes from public databases, and of these, three were soft corals and six were hard corals, and used a sponge genome (*Amphimedon queenslandica*) as the outgroup. Orthologous relationships were defined using OrthoMCL (Ver. 2.0.9; RRID: SCR_007839) (Li et al. 2003). We aligned one-to-one orthologs using MUSCLE (ver. 3.8.31; RRID: SCR_011812) (Edgar 2004) and eliminated ambiguously aligned regions using Gblocks (ver.0.9.1; RRID: SCR_015945) (Talavera and Castresana 2007). A phylogenetic tree was constructed using RAxML software (ver. 8.2.12; RRID: SCR_006086) (Stamatakis 2014) and employing the PROTGMMAAUTO model with an outgroup of the sponge genome. We estimated the divergence times using the MCMCtree (ver. 4.9) based on fossil calibration times. We used the café algorithm (ver. 4.2.1; RRID: SCR_018924) (Han et al. 2013) to estimate gene expansion and contraction throughout the coral evolution.

## Supplementary Material

evac132_Supplementary_Data

## Data Availability

All sequences generated in this study, including PacBio long reads and Illumina short reads, were deposited in the NCBI under BioProject PRJNA782406. The genome assembly and annotation files are available from the Marine Genome Information Center (http://www.magic.re.kr/assembly/MA00395) and the NCBI assembly accession GCA_024195265.1 (https://www.ncbi.nlm.nih.gov/assembly/GCA_024195265.1). The gene annotation files, including the functional gene and repeat gene, are available in the FigShare repository (https://doi.org/10.6084/m9.figshare.20400987.v1).
