# Supplementary material for: Comparative Genome and Evolution Analyses of an Endangered Stony Coral Species Dendrophyllia cribrosa Near Dokdo Islands in the East Sea
Source: Genome Biol Evol. 2022 Aug 26;14(9):evac132. doi: 10.1093/gbe/evac132 (PMC9455787; doi:10.1093/gbe/evac132)
Supplement: evac132_Supplementary_Data [file evac132_supplementary_data.zip › Dendrophyllia_GEB_Supplementary Figure_220810_revision.docx]

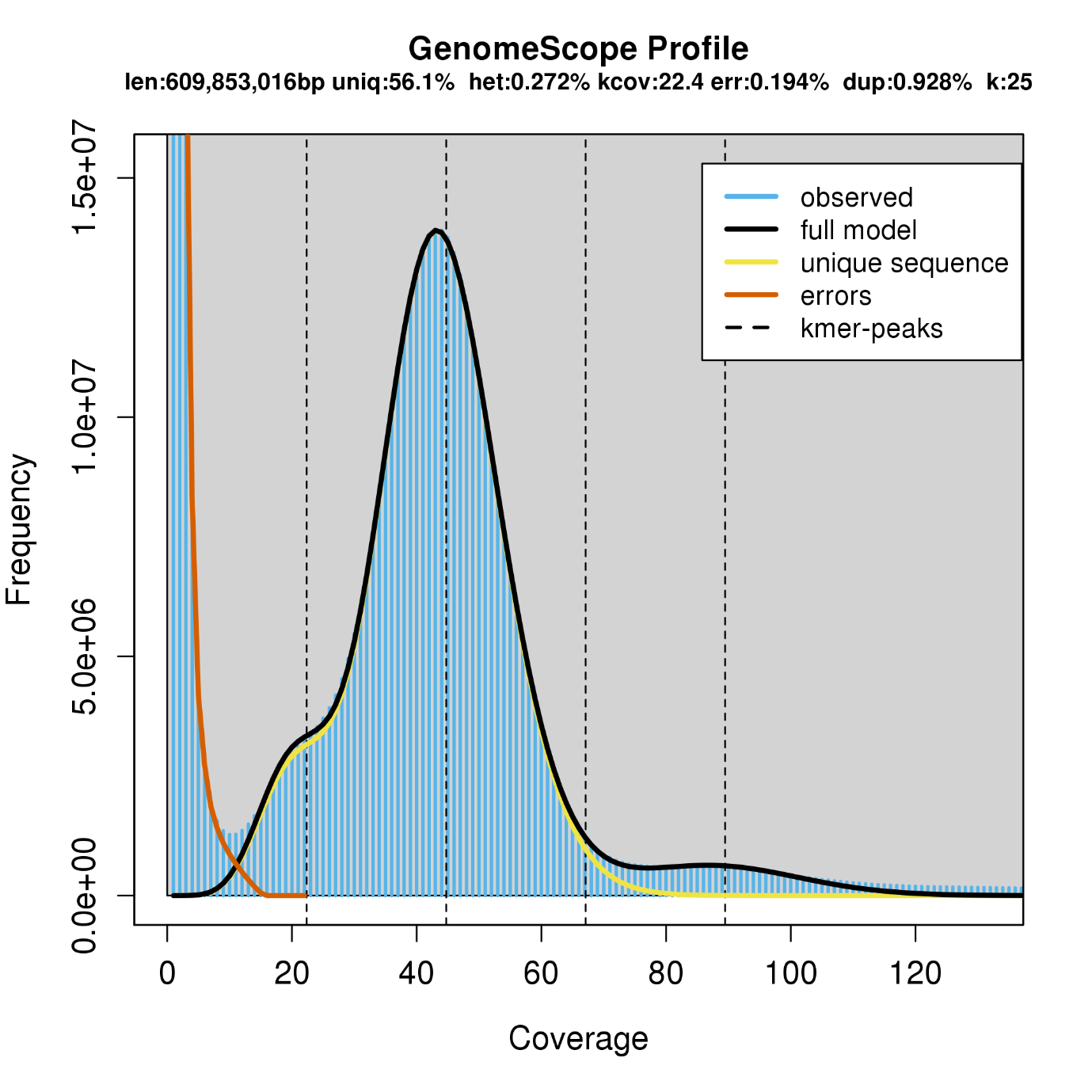


Supplementary Figure S1 Genome size estimation of *Dendrophyllia cribrosa*


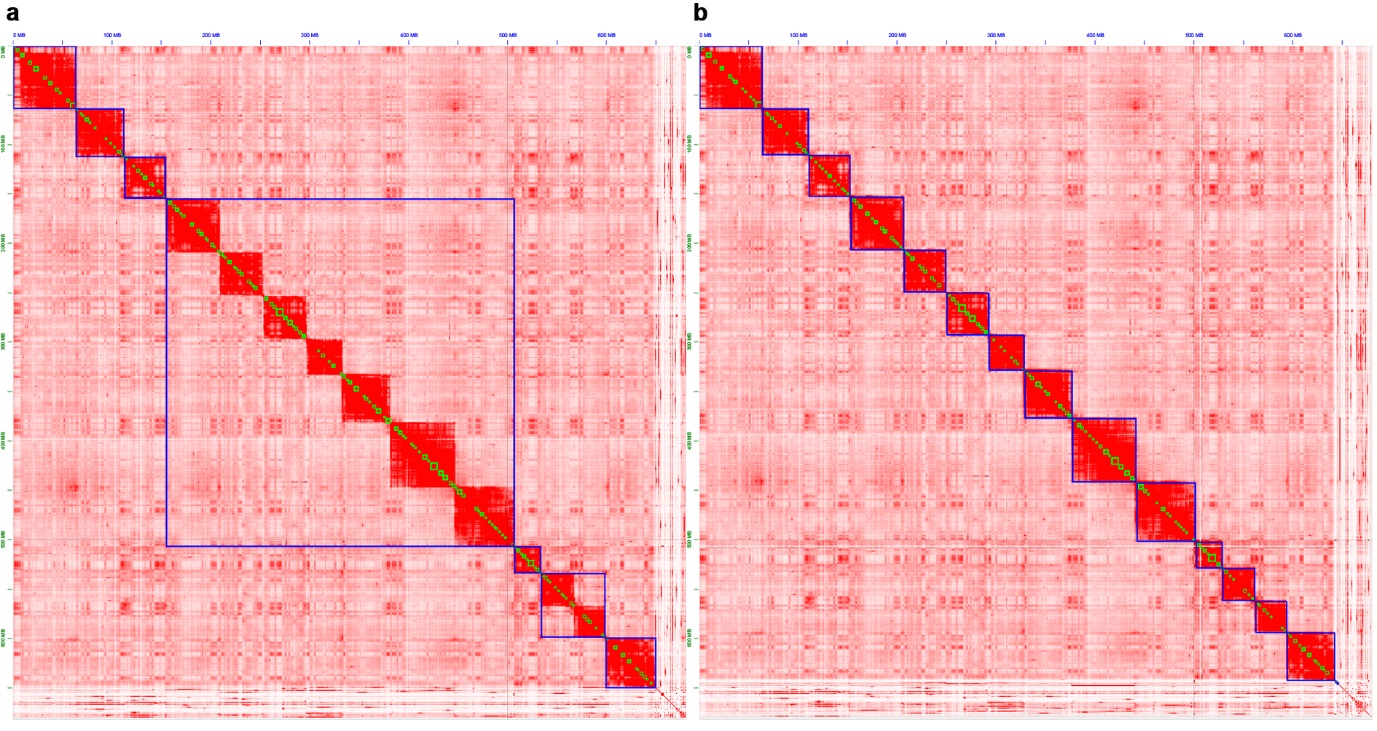


Supplementary Figure S2 Chromosome contact maps of *D. cribrosa*(a) before and (b) after manual curation
